# Agri-Food Wastes for Bioplastics: European Prospective on Possible Applications in Their Second Life for a Circular Economy

**DOI:** 10.3390/polym14132752

**Published:** 2022-07-05

**Authors:** Annamaria Visco, Cristina Scolaro, Manuela Facchin, Salim Brahimi, Hossem Belhamdi, Vanessa Gatto, Valentina Beghetto

**Affiliations:** 1 Department of Engineering, University of Messina, C.da Di Dio, 98166 Messina, Italy; cristina.scolaro@unime.it (C.S.); salim.brahimi@studenti.unime.it (S.B.); hossem.belhamdi@unime.it (H.B.); 2 Institute for Polymers, Composites and Biomaterials-CNR IPCB, Via Paolo Gaifami 18, 95126 Catania, Italy; 3 Department of Molecular Sciences and Nanosystems, University Ca’ Foscari of Venice, Via Torino 155, 30172 Mestre, Italy; manuela.facchin@unive.it; 4 Crossing S.r.l., Viale della Repubblica 193/b, 31100 Treviso, Italy; vanessa.gatto@crossing-srl.com

**Keywords:** biopolymer, biowaste, upcycle, circular economy, European Community trend, EU financed projects

## Abstract

Agri-food wastes (such as brewer’s spent grain, olive pomace, residual pulp from fruit juice production, etc.) are produced annually in very high quantities posing a serious problem, both environmentally and economically. These wastes can be used as secondary starting materials to produce value-added goods within the principles of the circular economy. In this context, this review focuses on the use of agri-food wastes either to produce building blocks for bioplastics manufacturing or biofillers to be mixed with other bioplastics. The pros and cons of the literature analysis have been highlighted, together with the main aspects related to the production of bioplastics, their use and recycling. The high number of European Union (EU)-funded projects for the valorisation of agri-food waste with the best European practices for this industrial sector confirm a growing interest in safeguarding our planet from environmental pollution. However, problems such as the correct labelling and separation of bioplastics from fossil ones remain open and to be optimised, with the possibility of reuse before final composting and selective recovery of biomass.

## 1. Introduction

Bioplastics are bio-based, biodegradable or compostable materials that constitute one of the most appealing alternatives for the substitution of fossil-based polymers, which can perhaps address the most pressing challenges facing Europe and the world for the protection of our planet (microplastic pollution and plastic islands in the oceans and seas) [1,2].

Traditional plastics come from petrochemicals and are classified as non-biodegradable materials. Oil resources cause enormous and concerning global pollution and constitute a limited resource [3]. The transition from a linear economy to a circular economy model is necessary to plan an efficient end-of-life treatment for plastics. The present and future use of new bioplastics must be based on eco-design and the development of materials not only for their usefulness but considering their reuse and recyclability afterlife [4,5]. Fossil-based plastics production has been based on a linear economy, leading to the environmental pollution we face today, which has only begun to be addressed globally [6].

The growing interest in safeguarding the world has led the scientific community to develop 100% bio-based and totally biodegradable plastics, such as polylactic acid (PLA), polybutylene succinate (PBS), poly ε-caprolactone (PCL), polybutylene adipate terephthalate (PBAT), polyhydroxyalkanoate (PHA), as well as bio-polyethylene (bio-PE), bio-polypropylene (bio-PP), bio-polyethylene terephthalate (bio-PET) made from bio-based building blocks [1].

**Figure 1:**
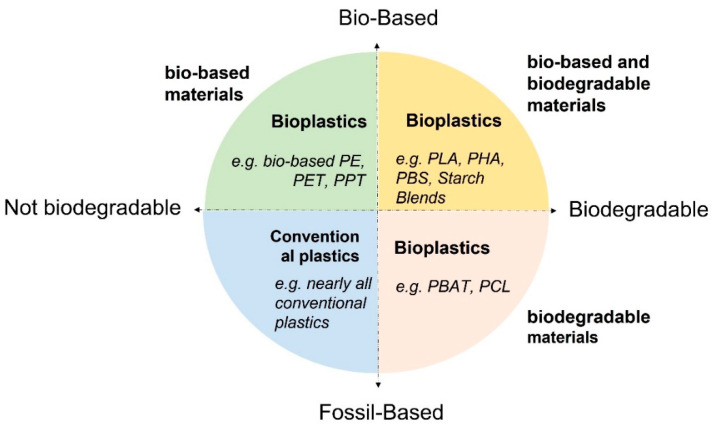
Examples of bio-based and fossil-based polymers are subdivided into biodegradable and not biodegradable types.

The biodegradability of bio-based plastics compared to fossil-based plastics is reported in Figure 1.

The data reported in Figure 1 shows an interesting feature: consumers generally tend to think that if a product is made from a biopolymer, it will be biodegradable, but this is not always true. For example, bio-PE made from sugar cane has physical-mechanical properties adequate to replace petrochemical-based products [2,7] as the latter is non-biodegradable.

On the contrary, the widely used PLA is an example of a bio-based biodegradable polymer, which nonetheless has physical-mechanical properties that are not always adequate to substitute fossil-based plastics. In fact, PLA is used to produce plastic bottles for water but has very low gas barriers and, therefore, cannot be employed for all drink vessels. Indeed, thanks to its biocompatibility, it is used for many applications such as bottles, cutlery, and biomedical implants [8,9,10].

Presently, biopolymers are disposed of in different ways according to their use or their labelling: plastic bags for organic waste are sent to industrial plants to make compost, PLA bottles are generally disposed of together with fossil-based polymers and are labelled as ‘7’ (other) and are typically not recycled. Other goods such as coffee cups may either end up with non-recyclable waste or be composted according to labelling. 

Plastic waste management and disposal pose a serious problem in terms of the recyclability of post-consumer plastics and the quality of materials made from recycled plastics. Labels on packaging may indicate chemical composition, whether they are recyclable or biodegradable, and, if so, under which conditions [11]. Unfortunately, today’s labels on packaging may vary depending on the industrial standard used for certification or geographical area of production. For example, different biodegradability standards exist within the EU, posing serious problems in end-of-life disposal and management, so presently, different standards are under investigation to harmonise labelling within the EU [11,12].

**Figure 2:**
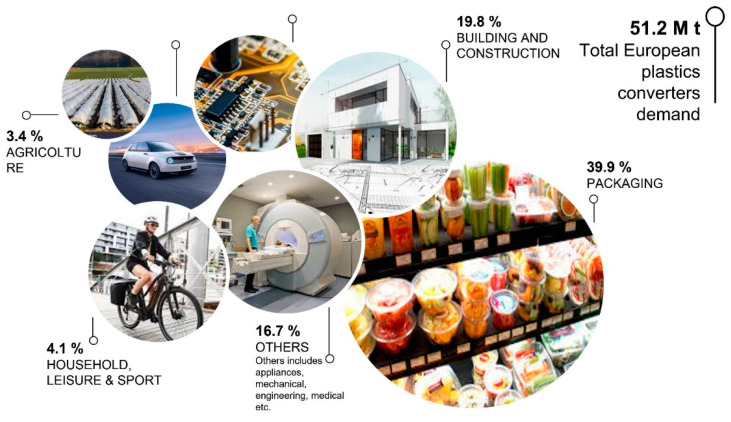
European fossil-based plastic market.

As is known, polymers are employed for the production of a wide variety of goods and products (Figure 2). Bioplastics are mainly used to replace consumer plastic, such as shopping bags, packaging, and disposable items that contribute to reducing fossil-based plastics disposal. Typical market segments for this are catering packaging (rigid and flexible) [12,13,14,15], medical-pharmaceutical sector [16,17], textiles and fibres [18,19], electronics [20], construction [21,22], automotive industry [23,24], agriculture and forestry horticulture [25,26,27,28], and coatings and adhesives [29].

The design of bio-based polymers is an eco-friendly alternative to fossil-based polymers, and the global market for green polymers, and bioplastic packaging materials is expected to reach $29.7 billion by 2026 [30]. Nevertheless, as mentioned above, these biopolymers exhibit low physical-mechanical characteristics compared to conventional synthetic polymers, which make them unsatisfactory for packaging applications [31]. Thus, the quest for new solutions to produce high-quality bio-based food and non-food packaging is still of great interest.

**Figure 3:**
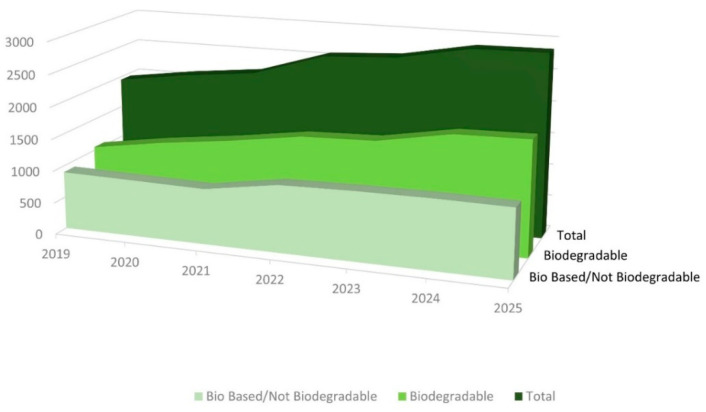
European bioplastic production capacity from 2019 and projections until 2025.

Biomass availability is a crucial point in the development of bioplastics since scant available feedstock and the monopoly of a few leading producers are controlling the biopolymer market and prices, strongly limiting their use. EU Bioplastics [32] reported in 2020 that the production capacity of biopolymers is expected to reach 2.87 Mt by 2025 (Figure 3), which is still too low in terms of market needs (360 Mt) [32]. The large gap between market demand and available bio-based plastics shows the need for alternative feedstock at sustainable costs to boost production. This is the reason why fossil-based plastics are still predominant (95%). Therefore, reduced fossil fuel consumption and the use of renewable and environmentally safe resources is likely to be the main advantage of bioplastics, together with the possibility of biodegradability at the end of their life.

Among the disadvantages of bioplastics, as listed by Di Bartolo and co-workers [1], a significant problem is their high production cost. Sohn and co-workers [33] highlighted that the future of bioplastics depends on the possibility of implementing new monomers that can help improve materials performance at sustainable prices. The difference in price between fossil-based and bio-based polymers is mainly due to the need for additional processes (for example, fermentation, purification, extraction) that should be improved to reduce additional costs.

It must also be considered that large areas of cultivation should be dedicated to the production of raw materials for the generation of bioplastics in addition to land for crops for food and animal feed.

In this review, the analysis of the state-of-the-art alternatives to reduce bioplastics production costs will be reported. Limiting the use of virgin bioplastics made from dedicated crops and using highly abundant and costless agricultural waste is an economically and environmentally sustainable way to reduce bioplastic costs. Starting from general considerations in the literature on bioplastic upcycling and composting/landfilling are discussed in Section 2. The pros and cons of the production, features and applications of biowaste for bioplastics are discussed in Section 3. Finally, works in the literature and the main trends and best EU practices on the use of agri-food waste (such as, for example, threshing from breweries, residual pomace from olives pressing, residual pulp from citrus fruits processing) as secondary materials to produce building blocks (monomers) or fillers for bioplastics, has been reported and discussed in Section 4.

As such, considering that the published reviews mainly focus on the recycling, production, application, and biodegradability of bioplastic as it is or composites (as we will see later), this analysis of the state-of-the-art in this review differs from to what was analysed in the current scientific literature.

## 2. Bioplastic Upcycling

As is known, polymer upcycling may be achieved by different recycling techniques: primary and secondary recycling are mechanical recycling, followed by tertiary or chemical recycling (feedstock recycling), and the last or quaternary recycling is an incineration process to recover energy. During mechanical recycling, thermoplastic materials are subjected to repeated processing steps that melt and stress the molten macromolecules. Thus, thermo-oxidative ageing occurs, which progressively shortens the life of the material [34].

For biobased non-biodegradable plastics, such as olefin biopolymers (i.e., bio-PE, bio-PP and biopolyamides (bio-PAs), the recycling process is equivalent to that employed for fossil-based polymers. According to literature studies and waste hierarchy, primary and secondary mechanical recycling are envisioned, followed by chemical recycling and energy recovery [29]. Bio-based/non-biodegradable bioplastics (like Bio-PET and bio-PE) maintain their mechanical properties for a reasonable number of recycled materials, then they may be chemically treated to recover the monomers used for re-polymerization [34,35].

Regarding biodegradable bioplastics, most of today’s post-consumer biodegradable biopolymers are used to produce compost, fertilizers, or biogas [36]. In the case of biopolymers, a distinction must be made between non-biodegradable and biodegradable polymers. It is possible to evaluate a recycling scenario for biodegradable biopolymers before their final biodegradation. In general, mechanical recycling is the simplest method from an economic, technological, and environmental point of view.

Maga and co-workers carried out a Life Cycle Assessment study (LCA) to evaluate the environmental impact of different recycling techniques starting from virgin PLA or blends of virgin and recycled PLA, compared to incineration. Depending on the recycling technology, savings in greenhouse emissions are 0.3 to 1.2 times higher compared to thermal treatment. The LCA study also evaluates the benefits derived from reduced fossil resource depletion, agricultural land occupation, photochemical ozone formation, terrestrial and aquatic eutrophication, and acidification. The study amply demonstrates that the recycling of PLA can contribute to a better environmental performance of PLA products in their life cycle. More information on LCA studies regarding different biodegradable biopolymers has also been reported [37,38,39].

Nevertheless, although the recyclability of biopolymers has been demonstrated, industrialization is still struggling [40]. It should be considered that for meaningful and cost-effective recycling of plastics and bioplastic, a critical mass of recyclable plastic is needed. In the case of biopolymers, this is difficult to obtain since their actual production still represents only a few units in terms of a percentage compared to the production of fossil-based plastics [41]. It has been estimated that 200 thousand tons are the minimum production quantity required to justify, from an economic point of view, the construction of a dedicated recycling plant processing around 5 and 18 kT/year of the polymer [34]. Considering that PLA production has reached approximately 200 kT worldwide in 2018 [42], these recycling facilities may soon be available. Nevertheless, this will be possible only if an adequate separation of PLA is carried out by converters and consumers. It is for this reason that the association of Plastics Recyclers Europe recently launched a call for the development of separate recycling streams for biodegradable plastics to improve waste management efficiency throughout Europe.

Few examples are reported in the literature relative to primary mechanical recycling of biodegradable aliphatic polyesters, and in particular of PLA, which is the most used biodegradable plastic [29,43,44]. The primary mechanical recycling of PLA takes place through the collection, washing and subsequent reprocessing of the material. This last step takes place with heat treatment and can compromise the properties of the material since thermomechanical degradation decreases the molecular weight of the polymer. This implies that recycled PLA is downgraded to a less demanding use. The 3D-printed PLA can only go through two recycles. A possible alternative to extend recycling could be to combine recycled PLA together with virgin PLA; however, the number of cycles is limited [29].

Examples of secondary mechanical recycling of bio-derived biodegradable post-consumer plastics show that compared to fossil-based plastics, biopolymers are less resistant to mechanical recycling and present greater problems, including thermal degradation, strong hygroscopicity, low glass transition value and low crystallization kinetics.

Furthermore, plastics are often loaded with fillers to specifically enhance certain properties. Both small percentages by weight (e.g., 0.5 wt%) [45] but also up to consistent percentages by weight (even 40–50 wt%) [46] are used. These fillers can be of biological origin as well as other types of reinforcing fillers. Additives and compatibilisers can also be added to the bioplastic blend and, in some cases, negatively influence mechanical recycling [44].

Hubbe and co-workers summarised literature works published between 2009 and 2021 in their review regarding the recycling of cellulose-reinforced PLA and PHB composites. These data show that given the nature of the fibres used, the material could be reprocessed up to 10 times in the best conditions, down to no more than three times in the worst conditions [40].

Overall, mechanically recycled post-consumer biodegradable bioplastics generally fail to guarantee good mechanical performance, so much is to be done to achieve this important target.

### 2.1. Blends of Bio-Based and Fossil-Based Polymers

Bio-based Plastics (B-bP) may be processed in combination with Fossil-based Polymers (F-bP) to achieve polymeric blends. In general, different parameters must be evaluated to obtain suitable physical-mechanical characteristics of the final polymeric blends [47,48]. This strategy may be adopted to reduce production costs improving market competitiveness, but it must be considered that polymeric blends’ recyclability may be compromised since increasing percentages of B-bP added to F-bP ones, or vice versa, may interfere with conventional recycling techniques [49].

Even in the best cases, when bio-based non-biodegradable plastics, such as bio-PP, bio-PE, and bio-PET are recycled together with their fossil counterparts, they can only be mechanically recycled or incinerated to produce energy [47]. If small quantities of PLA, even as low as 1 wt%, are mixed with PET in mechanical recycling operations, the thermomechanical properties of recycled PET are compromised. These data further highlight the complexity of bio- and fossil-based plastics disposal and management, requiring accurate separation by consumers [29].

Blends made primarily of biopolymers containing small quantities of fossil-based plastics may lose biodegradability and compostability [50,51,52].

The low miscibility of bio-derived, biodegradable plastics with other fossil-based plastics makes it difficult to recycle bioplastics; consequently, only very small quantities, around 2%, may be added not to compromise the final quantities of the product [43].

Overall blends containing bio- and fossil-based polymers, although less expensive, have not yet raised the interest of the industry, and further studies are necessary to reach the quality standards necessary for the scope.

### 2.2. The Composting/Landfilling of Bioplastics

Compost is the result of the bio-oxidation and humification of organic materials by macro- and microorganisms in the presence of oxygen. The gases generated during these processes in the soil (such as carbon dioxide (CO_2_) and/or methane (CH_4_) increase the GWP100 parameter (global warming potential), which indicates the number of greenhouse gases (GHG) produced. Landfill represents the final stage of bioplastics disposal and must be carried out according to specific regulations [36].

**Figure 4:**
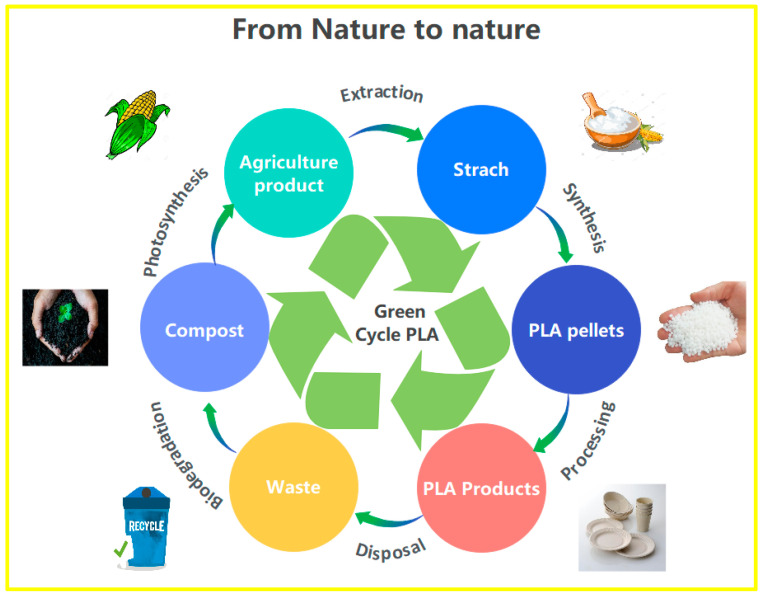
PLA’s life cycle.

The industrial composting process of PLA, for example, is done at 60 °C for approximately 30 days and produces only water and carbon dioxide with a slow rate of biodegradation [53]. Despite the advantageous property of polymers made from renewable resources, inappropriate end-of-life management can contribute to plastic pollution: bioplastics need to be collected and appropriately treated industrially and not left free as waste in the environment. Furthermore, contradictory scientific data exist in the literature on the biodegradability of PLA [54]. In soil or domestic composting machines, degradation can take up to a year with temperatures of 20 °C. In Figure 4, the life cycle of PLA (from nature to nature) is resumed. Similar considerations on the life cycle reported in Figure 4 can be extended to other plastics and biopolymers in general [55,56].

## 3. BioWaste for Bioplastic: Pros and Cons

### 3.1. From Agri-Food Waste to Biopolymers

According to the Food and Agriculture Organization (FAO) 2019 annual report, agri-food production worldwide was around 1.3 billion tons [57]. Food Losses and Waste (FLW) are serious economic and environmental problems, so FLW represents a global challenge due to its environmental impact [58]. FLWs contain high levels of vitamins, minerals, fibres, and proteins but do not meet food standards adequately and cannot be converted back into food. In these conditions, agri-food by-products become waste requiring disposal, possibly in landfills.

Alternatively, organic food waste can be transformed into materials with high added value. Consequently, there is a growing interest in the exploitation of this waste for other purposes, such as the production of bioplastics [59,60]. Thanks to their protein and polysaccharide content, indeed, eco-friendly bioplastics can be produced from renewable sources like casein [61], pear pomace and ricotta whey [62], watermelon [63], starch and sugarcane bagasse pulp [64], banana peel [65], lignin-cellulosic crop residues [66,67], soybean oil [68], grass pea [69], and algae [70]. The main polymers obtained by some FLWs are shown in Table 1.

The production of bio-based polymers from renewable sources and microbial synthesis has the advantages in that they are cheap, scalable, and have a minimal impact on the environment compared to the chemical synthesis of plastics from fossil sources (pros) [71].

The most common applications of bioplastics from agri-food waste are food packaging, hygiene products, coatings, scaffolds, absorbent and superabsorbent materials, as well as agriculture, automotive, construction, and medical materials [68,72]. Despite the development of advanced synthetic methods and their possible applications in the various sectors, commercial production is limited by production costs, which can be high to make them competitive compared to traditional plastics [58]. In addition, it will be necessary to implement production technologies specifically for bioplastics (such as electrospinning or 3D printing) [72].

Bioplastic production is also limited by other problems (cons):

- The life of the product is limited by the fact that natural biopolymers are susceptible to hydrolytic attack by water, which compromises their mechanical strength;
- The final biodegradation process can be another problem because it can be long and difficult and should be carried out industrially according to the required standards;
- The availability of virgin biomass and of agri-food waste is linked to the global variability of the various geographical areas based on the typical crops of the various countries. Sustainability is a primary criterion that conditions the choice of the type of starting material;
- The production of biopolymers requires high quantities of agro-waste: this is made difficult by the fact that there is still no well-organised separate collection of agri-food waste.

Considering a balance between the pros and cons listed thus far, it is immediately clear that there are more organizational difficulties and cons than advantages. However, factors such as the primary good of public health, and the protection of the planet, combined with the need to dispose of large quantities of waste produced, make it necessary to search for more adequate technologies and organizational methods than those currently existing for agri-food waste treatment. This also coincides with the necessity to solve the problem of plastic pollution.

Bioplastics are used in an increasing number of markets, from packaging, catering products, consumer electronics, automotive, agriculture/horticulture and toys to textiles and several other segments. Packaging remains the largest field of application for bioplastics with 47 percent (0.99 million tonnes) of the total bioplastics market in 2020. However, the portfolio of the application continues to diversify. Certain industries, such as automotive and transport, building and construction, or electric and electronics, remain on the rise with growing capacities of functional polymers. With a growing number of materials, applications, and products, the number of manufacturers, converters and end-users also increases steadily. Significant financial investments have been made into production and marketing to guide and accompany this development.

The factors driving market development are both internal and external. External factors make bioplastics an attractive choice. This is reflected in the high rate of consumer acceptance. Moreover, the extensively publicized effects of climate change, price increases of fossil materials, and the increasing dependence on fossil resources also contribute to bioplastics being viewed favorably. According to the latest Eurobarometer survey conducted by the European Commission, about 90 percent of European customers want to buy products with a minimal impact on the environment [73].

From an internal perspective, bioplastics are efficient and technologically mature materials. They can improve the balance between the environmental benefits and the environmental impact of plastics. Life cycle analyses demonstrate that bioplastics can significantly reduce CO_2_ emissions compared to conventional plastics (depending on the material and application).

Biopolymers currently have a higher cost compared to FbP polymers. Thus, in theory, it should be more convenient to use FbP. Nonetheless, there is a key factor that contributes to changing the rules of the game and economic perspectives—today’s consumers accept that they must pay more for products with higher sustainability. The higher prices of finished goods made with biopolymers drive more of a margin in the supply chain, thus neutralising the difference in the price of biomass-derived feedstocks and polymers. This fact is clearly demonstrated by plastic and bioplastic coffee capsules. Coffee capsules from fossil-based plastics have a price between 18.50/20.00 €, while bioplastic capsules have a premium price of about 25 € euro for 100 pieces.

This is a clear example that customers will pay higher prices for more sustainable products. Interestingly, even if biopolymers have higher prices, the higher price paid by final consumers for PLA capsules generates higher margins, making bioplastics profitable.

### 3.2. Use of Agri-Food Waste as Filler

As an alternative to the use of agri-food waste to produce building blocks for biopolymer synthesis, as discussed in Section 3.1, FLW can be used as filler in plastics or bioplastics. Typical bioproducts employed as fillers are lignocellulosic, starch, or fats from food waste in which the protein fraction is mostly relegated to low-value applications (e.g., animal food).

The interest in this direction is increasingly pushing researchers to study new methods to integrate fillers deriving from agri-food waste in different weight percentages with various thermoplastic and thermoset polymers.

Many studies have been carried out on the biodegradability of biocomposites from agricultural waste to push the industry to implement these materials on a large-scale [74,75].

Petroleum-based thermoplastics (such as HDPE-high density polyethylene, PP-polypropylene, and PVC-polyvinyl chloride) can be blended with fillers obtained from waste produced from the processing of grain, rice, sorghum, millet, walnuts, coconut, coffee, cotton, peanuts, sugar cane, flax, hemp, jute, straw, wood fibre, rice husk, wheat, barley, oats, rye, bamboo, kenaf, ramie, sisal, coconut fibre, kapok, raffia, banana fibre, pineapple leaf and papyrus fibre [74,75].

Petroleum-based thermoset polymeric matrixes (like epoxy resin, polyester, vinyl-ester, polybutylenes adipate-co-terephthalate (PBAT), polybutylene succinate (PBS) [76] or rubber [77] can also be blended with natural fibres. The latter can be useful for lightweight structural applications (wall insulation boards, roofing sheets, building boards, tiles) [78], and the evaluation of the best filler content is a key factor in the resulting mechanical and wear behaviour of such composites [79].

The scientific community at large agrees that the big plus of these types of composites is the low cost of agricultural waste used as filler. The moderate mechanical properties, and the long procedural steps to lower the fibre’s moisture content for the hydrophilic character of these materials, especially the lignin cellulosic, are important aspects to consider. This could be the consequence of the incompatibility between the hydrophobic thermoplastic matrix and the hydrophilic fibres [80]. Thus, the fibre–polymer interface interactions should be optimised for the improvement of the physical-mechanical features of such composites [81]. The intrinsic hydrophilicity of natural fibres deteriorates the bond between the polymer matrix and the fibre, thus compromising the final properties of the composite. In addition, the thermal instability of the natural fibres constitutes a drawback in the application and, therefore, in the temperature of use of composite materials reinforced with natural fibres [82].

However, the replacement of harmful petroleum-derived materials, such as polymers and additives, with more sustainable alternatives is the current trend in the modern polymer composite industry; therefore, everything must be done to overcome these limits.

Current applications in this direction are focused specifically on the use of bioplastics, rather than fossil-derived plastics, to have a 100% bioproduct filled with biowaste. The most common biopolymers are PHA (poly-hydroxy-alkenoate), PHBV (poly (3-hydroxybutyrate-co-3-hydroxyvalerate), PHB-co-HH (poly(3-hydroxybutyrate-co-3-hydroxyhexanoate), PBS (polybutylene succinate), PLA (polylactic acid), PCL (poly-ε caprolactone), PBAT (polybutylene-adipate-co-terephthalate).

Generally, PHA or PHBV are blended with biofillers such as agave fibre, wood flour, bran fibre, pineapple leaf fibre, jute, or hemp, or they are coated with olive leaf extract to observe the change in thermal and mechanical behaviour, water absorption, the degree of biodegradation, and to check the fibre–matrix interface with and without surface treatments to compatibilise the two phases to enhance their adhesion [83,84,85,86,87].

Below, we present the main findings of the publications on thermoplastic biopolymers mixed with agri-food waste as filler. These studies have highlighted the importance of the pre-treatment of agri-food waste and the optimization of both qualitative and quantitative chemical composition before the creation of mixtures with bioplastics to obtain the best physical-mechanical performance of the final products.

Giubilini and co-workers [88] studied mixtures of oat shells (8% by weight) with biopolymers PHB-co-HH to obtain a composite in which the presence of the filler slightly increases the mechanical properties in terms of stiffness without compromising deformability. A compatibiliser (silane) is necessary to increase the affinity between filler and matrix. They highlighted that the proposed blend represents a worthy valorisation of an agri-food industrial waste.

Nanni and Messori considered agri-food waste derived from wine. The fillers were extracts of seeds and wine lees mixed with PHB for different purposes. After the physical-mechanical characterization of the blends and their biodegradation analysis, the authors stated that these eco-friendly and cost-effective bio composites could find space in large-scale disposable applications where heat resistance and fast biodegradability are required simultaneously [89].

In addition, Chan and co-workers studied the natural weathering of a composite based on PHBV and wood flour (WF) at 50% by weight. The presence of WF slows down the degradation induced by humidity, and it controls the stability of the composites under natural atmospheric agents [90].

Another interesting study proposed the production of disposable cutlery made using three flours (grape, millet, wheat) mixed with xanthan and palm oil as possible replacement products for plastic materials. The authors created biodegradable spoons as an example of an ecological way to reduce the consumption of plastic [91].

PBS can be blended with hollow fruit bunch fibre (FFB), bamboo fibre (BF), rice straws and apple pomace (AP), typically with an extruder blending process [92,93,94,95]. Sometimes the fibres are treated as in the case of bamboo fibres, with an alkali treatment to remove the hydrophobic component from the surface of the fibres. Physical, morphological, and mechanical characterizations were carried out. The best percentages by weight of the filler were determined in order to not compromise the mechanical properties of the PBS. Particularly interesting results were obtained, with apple pomace added even up to 50 wt%. In this case, the PBS was crimped with maleic anhydride to increase interfacial adhesion with AP. It has been verified that the resilience of PBS can reach up to 150%, confirming that AP acts as a reinforcing filler [95].

The PLA can be reinforced with other biofibres such as jute for making linen, Kenaf, and lemongrass (besides bamboo) to have fully biodegradable green composites. Usually, the presence of the fibres enhances the thermal stability of the composites and their mechanical tensile/flexural modulus and strength [96,97,98]. Some authors considered PLA blended with PBS [99,100], with natural rubber [101], or PLA grafted with maleic anhydride to improve the fibre–matrix compatibility.

Hejna studied PCL filled with waste lignocellulose materials, such as brewers’ spent grain (BSG). This work also highlights the need for a pre-treatment of the filling due to the insufficient interfacial biopolymer/biological waste interaction. Thermomechanical treatment can change the chemical structure of the agro-waste; the filler size decreases, and the surface area increases with an improvement in compatibility and mechanical performance of the composite. The same author suggested that BSG and other natural materials (e.g., by-products of the coffee industry and other waste from various industrial sectors) may also be used as antioxidants to improve the oxidative stability of polymeric materials [102].

PBAT can be added with micro-crystalline cellulose (MCC) with coffee husk (CH) and rice husk (RH) with hemp fibre (HF) or grape pomace (GP) [103,104]. When the filler is added without a compatibiliser, smaller quantities can be used (for example, up to 20% by weight in the case of MCC) because, beyond this threshold, the presence of the filler can create cracks in the interface matrix of the fibre, which propagate premature fracturing of the the materials [105]. On the other hand, if compatibilisers (for example, silanes) are used, the higher wt% of filler can be used. Lule and co-workers prepared a biocomposite PBAT containing a high biofiller load (up to 40 wt%). In addition, in this case, there was an increase in the Young’s modulus and a reduction in production cost by 32%, making this composite competitive with the other polymers [106].

Some authors studied specific applications (of both animal and vegetable wastes) to be added to thermosetting matrices: Savio and co-workers [107] considered sheep’s wool, used as a matrix mixed with filling powders (pre-treated with various chemical steps and heat treatments) to produce panels for thermo-acoustic insulation in a circular economy perspective. Hidayat and co-workers [108] proposed the production of an eco-friendly and formaldehyde-free particleboard panel from agro-industrial residues (cassava stem, sengon wood waste, and rice husk) bonded with a natural rubber latex-based adhesive. The authors of [108] suggested that agricultural waste (corn cob, peanut peel, cassava peel, cocoa peel, plantain peel, rubber seed husk) can be added to natural gums to reduce consumption or improve product quality. Some authors studied natural rubber with cocoa pod husks and rubber seed husks in the amount of 40 wt%: the encouraging results of these studies indicate again that agricultural waste products have great potential also as fillers for natural rubber compounds [109,110].

A deep evaluation of the features of bio-based blends is necessary to implement the use of biofillers for new bioplastics as alternatives to traditional fossil-based ones. The physical-chemical-mechanical performances of traditional fossil plastics have been studied for a long time and implemented with various expedients since the last century, and the process has continued in recent decades. Given the new geopolitical trends for the protection of the planet and for a circular economy, these studies will be a starting point for the almost complete future replacement of fossil plastics with bioplastics. From the encouraging results of the scientific community, it is believed we are headed in the right direction to advance these sustainable methods.

## 4. The European Prospective

### 4.1. EU Policy on the Environment for a Circular Economy

The 21st century is affected by increasingly difficult climatic conditions that require the rethinking and redesign of the use, recovery, and recycling of polymeric materials with an integrated approach [111]. The circular economy is pushing toward radical changes in production and waste management, pursuing to reduce water, waste, and energy, contributing to achieving zero-waste production and environmentally sustainable management cycles [112,113].

In this scenario, the use of agro-industrial waste for the recovery of building blocks or fillers to produce biopolymers is particularly attractive. In fact, according to the definition reported in the European Action Plan, the Circular Economy is an economic system in which “the value of products, materials and resources is maintained in the economy for as long as possible, and the generation of waste minimised”, while Bioeconomy refers to “the production of renewable biological resources and the conversion of these resources and waste streams into value-added products, such as food, feed, bio-based products and bioenergy” [114]. The conversion of biomass by-products and waste into added-value products is intrinsically connected to the principle of circular economy and zero-waste production processes [115].

According to the waste hierarchy, biomass should first be employed to produce food and animal feed, then as secondary materials for high-value products, and finally for energy production or landfill [116,117]. This is in consideration of when agro-industrial by-products are useless and are disposed of in landfills as waste; they may become highly valuable and sustainable starting materials for different industries, increasing the availability of biomass.

This new approach within the principles of the circular economy brings about various significant benefits such as (i) increased availability of biomass; (ii) reduced consumption of fossil-based products; (iii) reduction of potential microbiological pollution; (iv) environmentally and economically sustainable solutions with the reduction of waste disposal; (v) reduction of greenhouse gasses due to the decomposition of agro-industrial waste.

Focusing on biopolymer production, some very interesting results have been published in the literature [27,32,118]. As previously mentioned, it must be underlined that although biopolymers are biodegradable and environmentally safe in many cases, their production poses an important problem regarding land use. In fact, the land used to grow renewable feedstock to produce bioplastics amounted to approximately 0.7 million hectares in 2020, corresponding to 0.015% of the global agricultural area used for pasture, feed, and food. This value is bound to grow significantly for bioplastics to become a sound, efficient alternative to fossil-based polymers.

The market demands new sustainable products, and society requires that these be produced in an environmentally friendly way. With the growing interest of consumers and large manufacturing groups in production chain sustainability, environmental products profile, and the use of renewable products versus fossil-based ones to reduce climate changes, are becoming relevant for a purchase choice.

Thus, dynamic producers that implement climate-related actions and environmentally sustainable products such as those financed by the EU will possess important leverage for the promotion of their products on the market. It is important to highlight that only a few technologies have been scaled up to date due to different obstacles. These obstacles can include quick biomass decomposition, high dehydration and transport costs. Funding from the European Union is of extreme importance to overcome these obstacles and validate the scalability of innovative processes and products.

The EU promotes the development of eco-friendly and economically sustainable products and processes both with legislation and financial incentives.

Specifically, the EU Programme and the sub-programme, Circular Economy and Quality of Life (Regulation 2018/1046 and 2021/783) [119,120], together with the sub-program Climate Change Mitigation and Adaption, Nature and Biodiversity, are committed to:

- Develop, demonstrate, and promote innovative products and processes to help reach the objectives of EU legislation and policy on the environment and to contribute to the implementation of Best Available Technologies (BAT) for the manufacturing industry and, therefore also for biopolymers production;
- Catalyse the large-scale deployment of successful technical and policy-related solutions for implementing the EU legislation and policy on the environment, integrating related objectives into other policies and into public and private sector practices, mobilizing investment, and improving access to finance. As Katri Kulmuni, Finland’s Minister of Finance, recently stated, “The goals of the Paris Climate Agreement will not be achieved by using public funds alone. We need the leverage from the whole economy, including financial and capital markets, to support the transition” [121];
- Support the implementation, monitoring, and enforcement of EU legislation and policy on the environment with the involvement of stakeholders at all levels (policymakers, public, private entities, and civil society). Pro-social and environmental attitudes can be encouraged by comprehensive public information and awareness and by adjusting social norms with the help of social media, stakeholders, and end-users.

The recovery and recycling of agro-industrial waste generates an important reservoir of valuable biomass, which can be useful to reduce the impact of fossil-based plastics and boost the production of bio-based plastics without land depletion. It must be said that, to date, there is no one correct solution but many different solutions that should network and collaborate to create an environment that intercepts the advantages of each solution, thus allowing to boost all solutions together.

### 4.2. EU Funded Projects on Waste Valorisation

The panorama of industrial solutions financed by the EU to tackle the problem of agro-industrial waste recovery and reuse for a “zero-waste economy” is extremely vast and comprises many different alternatives. The main EU-financed projects listed below foster the use of agro-industrial waste to produce high-value products for:

- Nutraceutics and cosmetics (see for example CIRCULAR AGRONOMICS H2020—Efficient Carbon, Nitrogen and Phosphorus cycling in the European agri-food System and related up- and down-stream processes to mitigate emissions [122]; VALOWASTE: Valorisation of waste streams from the agro-food sector [123],\; WASEABI Optimal utilization of seafood side-streams through the design of new holistic process lines [124]; LIFE OLEA REGENERA: Valorisation of biowaste resulting from the olive oil extraction process [125], WILDBERRY: Novel application targets and products derived from wild arctic berries [126]; UP4HEALTH: Sustainable and cost-effective production process for the upcycling of olive, grape and nut by-products into four natural and healthy ingredients for nutraceutical and cosmetic applications [127]; INGREEN: Production of functional innovative ingredients from paper and agro-food side-streams through sustainable and efficient tailor-made biotechnological processes for food, feed, pharma and cosmetics [128]; PolyBioSkin, High performance functional bio-based polymers for skin-contact products in the biomedical, cosmetic and sanitary industries [129];
- Energy (VIDA: Value-added innovation in the food chain [130]; UP-GRAD: Upgrading anaerobic digestion by cascade fermentation coupled with biogas-based biopolymer production [131]).
- Biogas (NoAW No-Agricultural Waste [132]; SolReGen: Using sunlight to create hydrogen from waste [133]);
- Biofertilizers (Circular Agronomics [134]);
- Biofuels (LIFE CoWaCo: Communal and organic waste conversion [135]; LIFE CIRCforBIO: A circular economy system for multi-source biomass conversion to value-added products [136]; LIFE STEAM: Green waste valorisation through innovative low-temperature STEAM explosion into advanced biofuel and agro-products [137]):
- Tanning agents (LIFE I’M-TAN: LIFE Innovative Modified Natural Tannins I’M-TAN [138]; LIFE BIOPOL: Production of leather-making biopolymers from biomasses and industrial by-products through Life-Cycle-Designed processes [139,140]).

It is interesting to note that projects financed by the EU have a few distinctive characteristics:

1. Starting from environmental problems related to agro-industry and waste minimization, the technological solutions allow the recovery of valuable biomass for biopolymer production, reducing the impact of fossil-based plastics and polymers;
2. All the technologies had not been implemented to industrial-scale previously due to high processing costs, insufficient knowledge of the technology involved and scale-up procedures.

### 4.3. EU-Funded Projects on Bioplastics from Agri-Food Waste

Focusing on bioplastics from industrial agri-food waste, different alternatives have been proposed and are described below.

TRANSBIO (Biotransformation of by-products from fruit and vegetable processing industry into valuable bioproducts) [141] was financed in 2011 for the valorisation of by-products from the fruit and vegetable processing industry using environmentally friendly biotechnological solutions (fermentation and enzymes) to obtain PHB, nutraceuticals and other platform chemical such as succinic acid. This project represents a valid solution to food waste recycling [142]. Partners involved in the TRANSBIO project developed cost-effective solutions to transform fruit and vegetable by-products into three valuable industrial products. Breakthroughs included finding bacteria that produce PHB from by-products like potato peelings and banana pulp and yeast strains that tolerate high levels of succinic acid. PHB produced was tested for packaging applications at the industrial level.

BARBARA is a three-year H2020 project that concluded in 2020, in which eight new materials were created through the valorisation of side-stream fractions and residues from agro-food waste into novel polysaccharides and functional additives [143]. These raw materials have been selected based on the advanced functionalities that provide for the polymeric matrixes. The polysaccharides were extracted, modified, and functionalised, and used as reinforcing additives with polyesters and polyamides for building and automotive sectors.

BIOREFINE-2G [144] concluded in 2017 and focused on closing the loop of biorefineries. Biorefineries produce ethanol, and as a major side- and waste-stream residual materials reach in pentose and lignin, which can be used for biogas and energy production but could be better valorised for high-value products. Partners of BIOREFINE-2G have developed new extraction processes to produce building blocks for bio-based thermoplastic polyurethanes (bio-TPUs) used as adhesives and coatings and polylactide (PLA)-copolymers which can be used as biodegradable packaging plastics.

BioCatPolymers [145] concluded in 2021 and had, as the main objective, to demonstrate a sustainable and efficient technological route to convert low-quality residual biomass to high, added-value biopolymers. The technology was based on an integrated hybrid thermochemical process combining the best features of both. The biological step consists of the conversion of biomass-derived sugars to mevalolactone (MVL). MVL can be then converted to biomonomers via highly selective chemocatalytic processes. BioCatPolymers is specifically aiming at the efficient and economical production of isoprene and 3-methyl 1,5-pentanediol (3MPD), two monomers with very large markets that can be further processed in the existing infrastructure for fossil-based polymers to produce elastomers and polyurethanes, respectively.

It is interesting to note that although many scientific papers have been published in the last years regarding the use of agro-industrial waste as fillers in combination with different biopolymers (as discussed in Section 3.2), very few projects have been funded, testifying to the limited applications of these technologies at industrial level.

### 4.4. EU-Funded Projects on Fillers from Agri-Food Waste

The main EU-funded projects in these projects and a summary of the environmental problems targeted are listed below, subdivided into different agri-food waste employed as the starting material:

- Lignin: Lignin is a waste produced by biorefineries, starting from wood and lignocellulosic crops, and is the second most abundant natural aromatic polymer after cellulose in terrestrial ecosystems. Depending on the isolation process and feedstock source, lignin can differ in structure, although such differences are not limiting factors for potential industrial applications [146,147]. Many research papers have been published on the use of lignin as an anti-inflammatory, anticarcinogenic, antimicrobial, prebiotic and antioxidant led to the use of lignin in many different sectors [148]. Lignins have been widely applied as raw materials to produce polymeric materials, carbon fibres, fuels, construction, and agriculture [149]. In line with the scope of this review, the EU-funded projects employing lignin as fillers for biopolymers are reported below;

SmartLi (Smart Technologies for the Conversion of Industrial Lignins into Sustainable Materials) [150] is a H2020 project financed in 2015 aiming to develop technologies for the recovery and recycling of technical lignins as fillers and building blocks for biopolymers. Technical lignins included in the study are kraft lignins, lignosulphonates and bleaching effluents, representing all types of abundant lignin sources. The technical lignins are not directly employable to produce biomaterials with acceptable product specifications. Therefore, pre-treatments have been developed to reduce their sulphur content and odour and provide constant quality. Thermal pre-treatments have been used to improve the material properties of lignin to be used as reinforcing filler in composites, while fractionating pre-treatments provide lignin which can be used as a plasticiser. Lignin may add value to composites also by improving their flame retardancy. Base catalysed degradation has been tested as means to yield reactive oligomeric lignin fractions for resin applications. Specifically, partial lignin degradation followed by downstream processing and further chemical modification leads to polyols employed for PU resin production. Full LCA, including a dynamic process, supported the study.

**Figure 5:**
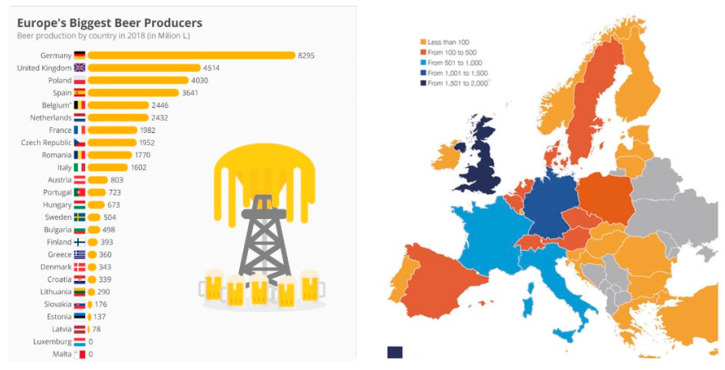
European Beer Producers (Eurostat 2020) and Breweries geographical distribution.

**Figure 6:**
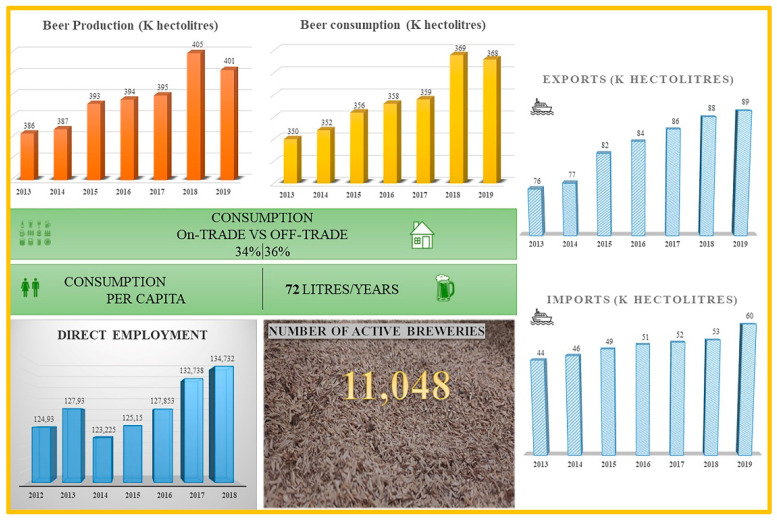
Economic data of EU beer production, consumption, import/export, and direct employment for year 2019.

- The project, SSUCHY: Promoting sustainable development with advanced bio-based composites [151], is fully integrated into the research program of the Bio-Based Industries (BBI) Joint Technology Initiative operating under Horizon 2020 and is focused on the production of advanced biobased materials starting from lignocellulosic feedstock used as filler. The project is oriented toward the development of multifunctional biodegradable and/or recyclable bio-based composites with advanced functionalities based on renewable resources. The main application of this project falls in transportation (automotive and aerospace) and a high-value market niche (acoustic and electronics).
- Brewery Spent Grain: Brewery spent grain (BSG) is a food waste product, the main side-stream from the beer brewing process representing 85% of the total by-products obtained from beer production. For every 1 hL of beer produced, 20 kg of BSG are generated [152,153]. According to Eurostat’s 2020 report, almost 39.5 MioL of beer were produced in the EU with incomes of 138.649 M€ in 2017, expected to reach 159.687 M€ by 2025 [154]. Over 6.4 Mt of brewery spent grain waste are produced yearly, generating a crucial management issue from an ecological and economic standpoint. These figures are nevertheless bound to increase, as explained below. An overall scheme of EU beer production capacities, geographical distribution, and economic data are provided in Figure 5 and in Figure 6 [155].

Landfill of biowaste is an unsustainable and expensive option; thus, most brewing industries have adopted disposal options for BSG that are within their financial and geographical reach, often favouring its use as animal feed, delivered almost for free (max. 15–20 €/ton) to farmers [156], with further transport costs of wet BSG, containing up to 80% water. Not only do breweries have a minimum/no return value from BSG given to farmers—this disposal of BSG is becoming less and less popular every year due to sanitary reasons (BSG has a shelf life of less than 48 h), leaving landfilling as a primary alternative, transforming a valuable by-product into waste, with management costs and environmental impact (every ton of BSG landfilled releases 513 kg CO_2_ equivalent of greenhouse gases). In EU about 70% of BSG is used as feed, around 10% goes to produce biogas, and the remaining 20% is landfilled [157].

In fact, according to a recent survey [158], it emerges that:

- An increasing number of farmers (>60% in Northern EU) are declining to take BSG as animal feedstock;
- None of the breweries own technologies for on-site storage of the spent grain;
- Failure of BSG regular disposal would force the brewery to halt production;
- BSG waste disposal costs are between 75–100 €/t in the EU.

Finally, the 2020 FDA draft rules acting under the *Food Safety Modernization Act* [159] foresee shutting down the brewery-to-farm transfer of spent grain to protect feed and dairy animals from potential health issues. It is likely that similar dispositions will soon be adopted also in the EU.

Friendly Knife [160] is the only financed EU project dealing with the recovery and recycling of BSG. This Horizon 2020 project studied the possibility of using BSG to make eco-friendly/biodegradable/disposable cutlery. The project intended to develop a new technology to produce single-use spoons, forks and knives, and possibly other items from brewer’s spent grain. These new products are said to be cheaper and more eco-friendly than the currently available disposable cutlery, although scarce information is available on the results of the project.

## 5. Conclusions

In this review, the various aspects that emerge from the analysis of the literature have been discussed, related to the recovery and recycling of agri-food waste both as precursors to produce bioplastics and as reinforcing agents for other bioplastics (mechanical and thermal) or as filler for bio-composites.

Their use appears to be precious considering the growing interest in the reduction of plastics produced from fossil sources in favor of bioplastics from vegetable sources to safeguard our planet from the great problems of environmental pollution that have emerged in recent times. The European directives are pushing the industry to adopt circular economy principles towards zero waste products, as demonstrated by the many scientific projects approved in this area and by the clear policies supported.

Agri-food waste is produced in large quantities and derives from many sources (from breweries, from the pressing of olives, from the production of fruit and vegetables, etc.). Consequently, the problem of their disposal arises both from the point of view of costs and means. However, precisely because these wastes represent a great added value for the substances they contain and which can be exploited, it is profitable to reuse and recycle them. The clear trend of the European Community towards a recycling and recovery policy, towards the replacement of plastic materials of fossil origin with vegetable-based materials, supports the studies carried out thus far in this direction. Furthermore, it is evident that the EU encourages other investigations to come, given there are still numerous problems remaining, ranging from the need to create ad hoc production plants (to improve the performance of bioplastics starting from the quality of the monomers) up to the correct recycling of these bioplastics with food waste fillers.

Further problems emerge from the study of the literature reported in this review regarding the recovery of bioplastics in relation to the possibility of reusing bioplastics with and without biofillers before their final composting. For example, Lamberti et al. estimate that plastic recycling may become a convenient alternative when at least 200 million tons of this plastic are recovered per year. Therefore, a specific system of “user-friendly” labels should be envisioned from the outset for their separation from the traditional fossil-based plastics with which they are commonly confused. One could also think of standardizing the labelling on a global level since it can vary according to the geographical area of production, as already mentioned.

Similar considerations can also be made regarding the recovery of biomass to be used as a biofiller. On an industrial level, it would be easier to recover the large quantity that is produced annually (which is of the order of millions of tons). At the private level of individual consumers, it is certainly much more difficult and complex to recover agri-food waste, as normally all organic waste is combined from separate collections.

Therefore, from this analysis of the state of the art, many points have emerged to work on in terms of the technical problems to be solved and the geopolitical limits to be faced for future environmental challenges of great importance.

## Figures and Tables

**Table 1:** Main polymer classes obtained from FLW. Adapted with permission from Ref. [58], 2 July 2022, MDPI AG.

		Polymer Class			
Waste Source		Polyesters	Polyurethanes	Polyamides	Polyolefins
Cuticle of fruits e vegetables	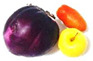	X			
Roots e tubers	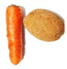	X	X		
Nutshell liquids	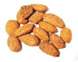	X			
Citrus fruits	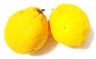	X	X		
Vegetable oils	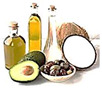	X	X	X	
Baked goods Cooked/dried sugary foods	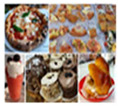	X	X	X	
Starchy biomass	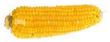	X	X	X	X
(Ligno) Cellulosic	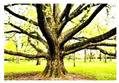	X	X	X	X

## Data Availability

The data presented in this study are available on request from the corresponding author.
